# The ninth International Conference of Anticancer Research, 6–10 October 2014, Sithonia, Greece

**DOI:** 10.3332/ecancer.2014.483

**Published:** 2014-11-18

**Authors:** Clare E Sansom

**Affiliations:** Department of Biological Sciences, Birkbeck, University of London, Malet Street, London WC1E 7HX, UK

**Keywords:** International Institute for Anticancer Research Conference, clinical chemotherapy, signal transduction, HOX genes, leukaemia, lymphoma, breast cancer

## Abstract

The ninth conference of the International Institute for Anticancer Research, held in Sithonia, Greece in October 2014, included over 700 abstracts presented in 79 separate sessions and featured a wide range of topics in basic and clinical cancer research. This report describes a small but representative sample of these sessions. It covers some recent developments in research into the basic signal transduction pathways involved in carcinogenesis; a special session on the role of homeobox genes in cancer development; and clinical sessions covering advances in breast cancer, haematological cancers, and chemotherapy.

## Background

The ninth conference of the International Institute for Anticancer Research (IIAR) was held from 6–10 October 2014 at the prestigious Porto Carras Meliton Hotel in Sithonia, Greece. This conference series, which began in 1995, was last held on the island of Kos in 2008 and now covers almost every aspect of basic and clinical cancer research. The 2014 meeting was held under the auspices of the IIAR and 24 other national and specialist cancer societies, mostly based in Europe, and it featured well over 700 presented abstracts arranged in a total of 79 separate sessions, a few of which are highlighted in this report. Some of these sessions brought together recent advances in the treatment of a particular tumour type, and others were specialist ones proposed and organised by individuals, or local or national interest groups.

The principal organiser of this conference series since its beginning has been the tireless John Delinasios, director of the IIAR and editor of the journal *Anticancer Research*. The breadth of the science presented at the meeting, along with high quality of the abstracts, large number of international delegates, ranging from students to emeritus professors, showed his commitment to building dialogue between basic and clinical researchers across the world.

## Advances in clinical chemotherapy

One of the first clinical sessions at the 2014 meeting took a new look at chemotherapy drugs. It began with a wide-ranging overview of the field by a long-term collaborator of Delinasios’, Franco Muggia of New York University Langone Medical Centre, New York, USA. He began by describing how endometrial cancer was rare among solid tumours in being well treated with surgery only, and how the relatively few patients who needed systemic treatment can benefit from well-established chemotherapy agents, including platinum-containing drugs and taxanes [1]. A series of phase III clinical trials in advanced or recurrent endometrial cancer has established carboplatin and paclitaxel, with or without doxorubicin, as the standard of care. However, the patient population is changing, with a higher percentage of older patients, obese patients, and patients with co-morbidities, and hence further trials are needed to determine the best treatment for these [1].

Ola Brodin from the Karolinska Institute, Stockholm, Sweden, gave an interesting presentation of the first clinical trial of a form of selenium as an anticancer agent. Selenium is an important trace element for humans, and an essential component of about 25 of our proteins, although high doses of it are toxic. This phase I trial, SECAR, set out to determine the maximum tolerated dose of sodium selenite. A small number of patients with confirmed carcinomas who were also receiving chemotherapy were given the selenium salt for two to four weeks. The results of this trial established a maximum tolerated dose of 10.2 mg/m^2^, and there seemed to be some indication of efficacy: 14 patients achieved stable disease, and there was one full remission [2]. A phase II trial is now planned.

Other talks in this session covered poly-chemotherapy regimens for exocrine pancreatic cancer, which has an extremely poor prognosis, and the use of ^90^Yttrium in brachytherapy.

## Oncogenes, tumour suppressors, growth factors, and signal transduction

Several wide-ranging and well-attended sessions covered the signal transduction pathways that are involved in cancer development and the mutation landscape of the genes concerned. One began with Dean Felsher of Stanford University, CA, USA introducing the concept of ‘oncogene addiction’, which suggests that inactivating some but not all oncogenes will always lead to cellular senescence or apoptosis. He proposed that the Myc would have these properties, and described a transgenic mouse model developed by his group in which this gene can be reversibly inactivated. Results from this model suggest that a healthy immune system is necessary for Myc inactivation to cause tumour regression [3].

Roche-Philippe Charles from Helen Diller Family Comprehensive Cancer Centre, San Francisco, California, USA described the mutational landscape of anaplastic thyroid carcinoma (ATC), which is the most aggressive form of thyroid cancer. Only about 5% of thyroid tumours are classified as anaplastic, but these are hard to treat and responsible for many more cancer deaths than papillary or follicular thyroid cancer. The V600E mutation of the proto-oncogene BRAF is found in 25% of anaplastic thyroid cancers, where it is frequently found in combination with mutations in the catalytic subunit of PI3 kinase. Transgenic mice bearing mutations in both these genes rapidly develop dense cellular thyroid carcinomas that dramatically increase the volume of the thyroid. Charles suggested that this mouse model forms an excellent pre-clinical platform for testing drugs against ATC or other tumours that bear these mutations [4].

Arun Seth from Ontario Institute of Cancer Research, Canada, who co-chaired the session with Felscher, described a mouse model of bone metastasis from breast cancer in which mouse flanks were implanted with healthy bone taken from women undergoing hip replacements as well as with human breast cancer cells. These mice are being used to evaluate potential treatments for metastatic breast cancer, as a quick test of whether an individual breast tumour is likely to metastasise, and to compare gene expression in metastatic and indolent breast tumours. Seth and his co-workers discovered that insulin-like growth factor binding protein 7 (IGFBP7) was down-regulated only in metastatic breast tumours [5].

Several other speakers, including Warren Thomas from Beaumont Hospital, Dublin, Ireland, discussed the clinical application of studies of signal transduction in cancer. Thomas gave an interesting talk about mesothelioma, an aggressive tumour that is linked to asbestos exposure and that is expected to peak in incidence in the UK and Ireland in the 2020s as the exposed population ages. He presented survey results that showed that females with this condition have, on an average, a longer survival time after diagnosis than males. This suggested a link between this cancer and oestrogen signalling, and Thomas found that high levels of one oestrogen receptor beta isoform, ERβ2—which cannot bind oestrogen—were correlated with good median survival. High levels of the steroid receptor co-activator SRC-2 and of p16 were also associated with a relatively good prognosis in these patients [6].

Further presentations covered personalised therapies for lung cancer and an association between the BRAF^V600E^ mutation in colorectal cancer, right-sided tumour presentation, and anaemia.

## HOX genes and cancer

One special session, organised by Richard Morgan and Claire Aukim-Hastie of the University of Surrey, Guildford, UK, concerned the relationship between homeotic or HOX genes and the cancer. Morgan began the symposium by giving a brief introduction to the function of the HOX genes, which encode transcription factors, and their role in cancer development. His first slide featured an image known to all advanced students of biology: an Antennapedia fly mutant showing a pair of legs growing in the place of antennae: this mutation is caused by a single mutation in one such gene. This illustrates the most well-known function of these genes, i.e., their involvement in defining the roles and identities of cells and tissues during development. However, they are also involved in regulating cell proliferation and survival, which are among the ‘hallmarks’ of cancer. Humans have a total of 39 HOX genes, classified into four groups, and many of these have been implicated in the development of one or more types of cancer.

Morgan then described some of his recent work with HOX genes as targets and biomarkers in malignant mesothelioma, an asbestos-related tumour that most often affects the outer lining of the lungs. Some of the HOX genes in the human genome seem to function largely as oncogenes, others as tumour suppressors, and the role of others is still unclear. He has focused on the interaction between these genes and the pre-B cell leukaemia transcription factors (PBX) proteins, which act as co-factors; blocking the dimerisation of a HOX protein and its co-factor prevents its activity. His group have designed a six-residue peptide now named HXR9 that disrupts the interaction between HOX and PBX proteins and prevents HOX activity. A second peptide, named CXR9 (the control peptide), differs from the first at one amino acid position only but does not prevent dimerisation. Mesothelioma cells express high levels of two HOX genes, HOXA4 and HOXA5; when HXR9 is added to block HOX activity, mesothelioma cells but not normal mesothelial cells undergo apoptosis [7].

Keith Hunter from the University of Sheffield described the role of HOX genes in head and neck squamous cell carcinoma (HNSCC). About 600,000 people worldwide are diagnosed with this tumour each year, and it causes 350,000 deaths. The molecular basis of the disease is very heterogeneous, with many genes implicated in at least some cases, but only p53 is almost universally mutated and only one targeted therapy, an epidermal growth factor receptor (EFGR) inhibitor, is available for these patients. Hunter and his colleagues identified differentially expressed genes from a panel of HOX and HOX-related genes in cell cultures from normal oral keratinocytes, pre-malignant tissue, primary HNSCC tissue, and metastases. Several of these genes were differentially expressed between tumour and normal tissue, and two, HOXB9 and HOXD10, were highly expressed in pre-malignant and primary tumour tissue but not in metastases or normal cells. These two transcription factors appear to promote cell proliferation and migration, but decrease invasion. Genes directly regulated by HOXD10 include AMOTp80 and a micro-RNA, miR146a, and knockdown of these genes inhibits the proliferative phenotype in HNSCC cells [8]. Hunter concluded by suggesting that the protein AMOTp80 might be a valid therapeutic target for this tumour type.

Manuel Penichet from the University of California Los Angeles, California, USA, described his group’s analysis of the expression and role of HOX genes in multiple myeloma. This cancer of monoclonal plasma cells in the bone marrow is currently responsible for about 75,000 deaths a year worldwide. Penichet and his colleagues tested the expression levels of HOX genes and the cytotoxic effect of the ‘anti-HOX’ peptide HXR9 in malignant B-cell lines including four multiple myeloma lines; one of these, U266, is a ‘bullet-proof’ myeloma line that is particularly resistant to apoptosis. Several HOX genes were differentially expressed in one or more of these cell lines, and HXR9 induced apoptosis in all lines, indicating that they were all dependent in some way on HOX expression. An antibody-avidin fusion protein, ch128.1.Av, which is specific for the human transferrin receptor 1 (CD71) is also toxic to malignant B cells as it deprives them of iron. The cytotoxic effect of HXR9 in myeloma cells was found to be enhanced if it was combined with this protein, suggesting that both HOX proteins and the transferrin receptor might be valid drug targets for multiple myeloma [9].

Acute myeloid leukaemia (AML) is a genetically heterogeneous disease, and its treatment, which consists largely of standard chemotherapy drugs, has scarcely changed in the last 30 years. Altered expression of HOX genes is, however, a hallmark of this disease, and Alexander Thompson of Queen’s University Belfast, Northern Ireland, UK, described the development of a HOX-based screen for candidate drugs for this condition. Many cytogenetically normal AML cells—that is, cells with no chromosomal translocations—express high levels of genes in the HOXA cluster, and patients with this type of AML and low expression of HOXA genes tend to have a better prognosis than similar patients with high HOXA expression. Acute myeloid leukaemia can be established in a mouse model by overexpressing HOXA9 and another homeobox protein, Meis1. A methylcellulose screen of this mouse model and cell lines taken from 351 cytogenetically normal AML patients, plus mouse and human controls, identified several small molecules already registered by the US Food and Drug Administration (FDA) that were able to change the HOX gene expression profile of leukaemia cells close to that of normal myeloid cells. Thompson described entinostat, a histone deacetylase inhibitor that is undergoing clinical trials for the treatment of Hodgkin lymphoma, breast and lung cancer, as the ‘most interesting’ of all these, and he suggested that direct targeting of overexpressed HOX proteins might be an appropriate strategy for treating AML and other blood cancers [10].

Other talks in this session described the role of HOX genes in breast and ovarian cancer and malignant melanoma, and discussed their role in pluripotent stem cell differentiation.

## Advances in haemato-oncology

Two clinical sessions held on the final day of the 2014 meeting, Friday 10 October, covered recent advances in breast cancer and haematological malignancies respectively. The haematological session began with a wide-ranging overview of the current treatment options for chronic myeloid leukaemia (CML) by Binay Shah of St. Joseph Regional Medical Centre, Lewiston, Idaho, USA. About 15–20% of adults diagnosed with leukaemia will have CML; the median age at diagnosis is 55, with most patients presenting in the chronic phase of the disease. On the cellular level, it is almost always characterised by the so-called ‘Philadelphia chromosome’ translocation between chromosomes 9 and 22, resulting in the aberrant tyrosine kinase Bcr-Abl. The prognosis for CML patients improved dramatically in the early 2000s when the first inhibitor of this kinase, imatinib, entered clinical use. Shah described several landmark clinical trials that led to the addition of two further tyrosine kinase inhibitors, dasatinib, and nilotinib, to the range of first-line therapies available for this condition. The choice of initial therapy remains unclear: dasatinib and nilotibib produce better cytological responses in most patients than imatinib, but are less well tolerated, and imatinib is also cheaper. The two newer drugs are therefore probably better initial choices unless a patient has certain co-morbidities or unless cost is a serious concern. Patients can be switched to a second kinase inhibitor if they fail the first; those who carry the T315 mutation and those who fail two can now be offered a promising new drug, the protein translation inhibitor omacetaxine [11].

Tamar Tadmore from Bnai-Zion Medical Centre, Haifa, Israel, described the aetiology and treatment of Richter syndrome, a rare complication of B-cell chronic lymphocytic leukaemia (CLL). Most patients diagnosed with this condition experience long remissions, but in about 5% of patients the leukaemia cells become transformed into an aggressive lymphoma: this is Richter syndrome, and it carries a poor prognosis. The most common transformation is into a diffuse large B cell lymphoma (DLBCL). A small minority of cases involve transformation into Hodgkin lymphoma, in which case the disease is known as Hodgkin’s variant Richter syndrome (HvRS). Diffuse large B cell lymphoma or Hodgkin lymphoma can also develop separately in patients with CLL, and this has a very similar aetiology and prognosis to so-called ‘true’ Richter syndrome.

Tadmore described a series of 119 patients diagnosed with Richter syndrome at 12 centres within Israel between 1996 and 2010. Sixty percent of the patients were men, and 89% were of Jewish origin (compared to 75% of the population of Israel as a whole). Patients with DLBCL have a worse prognosis than those with HvRS; Tadmore found the five year survival rates of the two cohorts to be 8 months and 39.5 months following diagnosis of Richter syndrome respectively. Both Richter-related DLBCL and HvRS are treated with the same chemotherapy regimens as diffuse large B cell lymphoma and Hodgkin lymphoma, but there is evidence that at least some patients with DLBCL will benefit from the addition of a monoclonal antibody, particularly rituximab [12].

Cecilia Evangelisti from the University of Bologna, Italy, described the potential of drugs targeting the sphingolipid pathway for treating T-cell acute lymphoblastic leukaemia (T-ALL). This type of the disease accounts for about 15% of paediatric and 25% of adult leukaemia cases worldwide; many patients can be relatively well treated with drugs, but novel, less toxic therapies are still needed. A signalling pathway involving a class of membrane lipids known as sphingolipids is known to be crucial in determining the balance between cell death and cell survival. The sphingosine kinases SK1 and SK2 play important roles in this pathway by phosphorylating sphingosine to its 1-phosphate (S1P). Evangelisti explained that both T-ALL cell lines and cells taken from patients with this condition undergo apoptosis when treated with a non-selective SK1/SK2 inhibitor. However, a minority of these cells become resistant to the inhibitor through activating the endoplasmic reticulum stress/unfolded protein response, which leads to a pro-survival autophagy. In contrast, treating the same cell lines and primary cells with the specific SK2 inhibitor ROMe induce uniform cell death through autophagy [13]. She suggested that specific inhibitors of SK2 might be potential therapies for this type of leukaemia.

## Advances in breast cancer

Breast cancer is as heterogeneous a disease as chronic or acute leukaemia, and the molecular profile of a patient’s cancer may change over time. In a session devoted to the aetiology and therapy of all types of breast cancer, Marius Raica from Research Centre Timișoara, Timișoara, Romania described his work in determining the molecular profiles of primary breast tumours and corresponding lymph node metastases. Breast tumours are currently classified into five main molecular subtypes–luminal A, luminal B, basal, HER-2 positive and ‘other’ (or sometimes ‘normal-like’)–based on expression levels of molecular markers including, but not restricted to, the oestrogen, progesterone, and HER-2 receptors. Until now, most measurements of these expression patterns have been made in primary tumours only.

Raica then described experiments to stratify both primary tumours and matched lymph node metastases and to determine the extent to which both the expression pattern and the derived subtype changed or remained stable in individual patient. He found changes in expression pattern that led to a re-classification in about a quarter of all patients tested. The HER-2+ subtype was the most stable, with few conversions between HER-2+ status in primary tumours and a different subtype in the lymph node metastases. In contrast, the luminal A subtype was the least stable, with many luminal A primary tumours being re-classified as luminal B or even basal in the metastases. He concluded that a re-evaluation of the molecular profile of lymph node metastases may be useful both in modifying the therapeutic strategy for a subset of current patients, and in identifying new targets and potential therapies [14].

Further talks in the same session discussed the role of oestrogen blocking and tailored chemotherapy in pre-menopausal patients with breast tumours at high risk of recurrence, and the use of polymorphisms in the gene CYP19A1, which codes for aromatase, in predicting the outcome for post-menopausal patients treated with the aromatase inhibitor letrozole.

## Conflicts of interest

The author has no relevant conflicts of interest.
